# β-acetoxyisovaleryl alkannin (AAN-II) from *Alkanna tinctoria* promotes the healing of pressure-induced venous ulcers in a rabbit model through the activation of TGF-β/Smad3 signaling

**DOI:** 10.1186/s11658-021-00278-5

**Published:** 2021-07-31

**Authors:** Xiao Yang, Weijing Fan, Renyan Huang, Guobin Liu

**Affiliations:** 1 Peripheral Vascular Disease Unit of the TCM Department, Shuguang Hospital, Shanghai University of Traditional Chinese Medicine, 201203 Shanghai, China; 2 Disease Unit of the TCM Department, Shuguang Hospital, , Shanghai University of Traditional Chinese Medicine, Zhangheng Road No. 528, Pudong New Area, 201203 Shanghai, China

**Keywords:** Skin ulcer, AAN-II, TGF-β1/Smad3 signaling, Inflammation, Tissue repair

## Abstract

Alkannin-based pharmaceutical formulations for improving wound healing have been on the market for several years. However, detailed molecular mechanisms of their action have yet to be elucidated. Here, we investigated the potential roles of AAN-II in improving the healing of pressure-induced venous ulcers using a rabbit model generated by combining deep vein thrombosis with a local skin defect/local skin defect. The extent of healing was evaluated using hematoxylin and eosin (HE) or vimentin staining. Rabbit skin fibroblasts were cultured for AAN-II treatment or TGFB1-sgRNA lentivirus transfection. ELISA was used to evaluate the levels of various cytokines, including IL-1β, IL-4, IL-6, TNF-α, VEGF, bFGF, TGF-β and PDGF. The protein levels of TGF-β sensors, including TGF-β, Smad7 and phosphor-Smad3, and total Smad3, were assayed via western blotting after TGF-β knockout or AAN-II treatment. The results show that, for this model, AAN-II facilitates ulcer healing by suppressing the development of inflammation and promoting fibroblast proliferation and secretion of proangiogenic factors. AAN-II enhances the activation of the TGF-β1-Smad3 signaling pathway during skin ulcer healing. In addition, the results demonstrate that AAN-II and TGF-β have synergistic effects on ulcer healing. Our findings indicate that AAN-II can promote healing of pressure-induced venous skin ulcers via activation of TGF-β-Smad3 signaling in fibroblast cells and provide evidence that could be used in the development of more effective treatments.

## Introduction

Venous leg ulcers (VLUs) are a common vascular condition that can cause a high socioeconomic burden. They are an advanced clinical manifestation of venous insufficiency. Their incidence is increasing along with the increased incidence of obesity and diabetes. They are also associated with aging. In the U.S. over 65 population, the incidence is 1.5–3 people per 1000, meaning 0.5–2 million people annually [1, 2]. Changes in venous hemodynamics often cause an increase in venous pressure, contributing to some extent to the difficulty of managing the ulcer. Thus, VLUs are generally secondary to venous hypertension. They are known to arise due to a series of complex cellular humeral events and potentially also some genetic factors [3].

Notably, venous ulcers often have recurrent features, implying that a better understanding of the underlying pathophysiology could improve treatment [4]. At present, there are many methods for making ulcer models, but there is no well-accepted one, which hinders the study of the underlying mechanisms, pathogenesis and response to drugs. There are at least 3 types of animal model: the mesenteric venule occlusion model, arterio-venous fistula model and large vein ligation model, in which venous hypertension is respectively induced by acute venular occlusion, placement of a chronic arteriovenous fistula, and ligation of several large veins [5]. For our study, we established a model using local skin defects based on the establishment of a deep vein thrombosis model in the lower limbs of rabbits.

Current therapeutic approaches include advanced wound dressings, antibiotics and surgery [2]. Some ongoing clinical trials are investigating systemic pharmacological agents as adjuncts to venous ulcer healing. Among these agents, herbal therapy with marked healing and anti-microbiological effects shows promise [6].

The root of *Alkanna tinctoria* contains compounds with antimicrobial, anti-inflammatory and antileishmanial activities. Extracts from it have been used as a botanical drug for ulcers, inflammation and wounds since ancient times [7]. One such extract is alkannin, which contains four active compounds: β, β-dimethylacryl alkannin (AAN-I), acetoxyisovaleryl alkannin (AAN-II), acetyl alkannin (AN-III) and alkannin (AN-IV). It has antitumor effects based on multiple-target mechanisms, including crosstalk with an alkylating agent, DNA and protein; effects on ROS levels; and influence on multiple signal pathways [8]. More recent reports demonstrate that alkannin can suppress lung histopathological changes and relieve the lipopolysaccharide-induced inflammatory injury [9, 10].

Of note, alkannin can also suppress the function of activated immune cells in psoriasis [28–30] and the four bioactive components have gained recognition as potential ingredients in dermal healing substances [11]. AAN-I has been reported to contribute to reepithelization of wounds through promotion of cell proliferation, migration and vessel formation [12, 13]. AN-IV can inhibit UVB-induced apoptosis via regulation of HSP70 expression in human keratinocytes [11]. The IC_50_ values of the four alkannins were recently determined for human dermal cells and shown to be significantly different [14]. Notably, AAN-II shows a strong healing effect and can significantly suppress H_2_O_2_-induced cellular senescence, possibly through upregulation of the expressions of collagen I and elastin in human dermal fibroblasts or keratinocytes [15]. These findings and advances on the effects of alkannin on promoting wound healing encouraged us to investigate the roles of AAN-II in venous ulcer healing.

Although alkannin-based pharmaceutical formulations for improving wound healing have been on the market for several years and their roles in wound-healing have been extensively demonstrated [16], the detailed molecular mechanisms have yet to be elucidated. Therefore, we investigated the role of AAN-II in improving the healing of pressure-induced venous ulcers using our rabbit model.

## Martials and methods

### Pressure-induced venous rabbit model

We purchased 30 adult female New Zealand rabbits from the Shanghai Laboratory Animal Center of the Chinese Academy of Sciences and randomly divided them into three groups: control (no ulcer), ulcer without treatment, and ulcer with AAN-II treatment (acetoxyisovaleryl alkannin; CAS No. 69091-17-4; purity 99 %; C_23_H_26_O_8_; MedChemExpress, USA). The treatment dosage was 20 mg/kg body weight.

The ulcer was established by causing a local skin defect based on the deep vein thrombosis model. The wound tissues were sampled for relevant tests on days 7 and 14. The physiological and behavioral characteristics of animals in each group were recorded daily. The ulcer area was calculated as its length times width times 1/4 π. All animal studies were approved by the Institutional Animal Care and Use Committees of Shuguang Hospital Affiliated to Shanghai University of Traditional Chinese Medicine (Approval No. 20,180,122 on March 1 2018) and performed in adherence with the Basel Declaration and the institutional guidelines for the care and use of animals.

### Hematoxylin-eosin (HE) staining and immunohistochemistry (IHC)

HE staining of formalin-fixed paraffin-embedded (FFPE) tissue sections were performed using the standard protocol. After HE staining, the FFPE sections were histopathologically evaluated and assessed for collagen deposition.

A second batch of FFPE sections was used for IHC examination with a primary antibody against vimentin. After deparaffinizing, the sections were permeabilized in a citrate buffer solution, microwaved for 10 min, washed with phosphate-buffered saline (PBS), then put in 3 % H_2_O_2_ for 15 min to block endogenous peroxidase activities. After washing with PBS, the sections were incubated with goat serum for 30 min, then overnight with the anti-vimentin primary antibody (1:200 at 4 °C). The subsequent steps were performed following the protocol for the secondary biotinylated antibody kit (Zhongshan Biotech, China). Histological images were taken with a digital-sight imaging system (Nikon Corporation, Japan).

### Cell viability assay

Cell viability was measured using a Cell Counting Kit-8 (CCK-8) according to the manufacturer’s protocols (Dojindo Laboratories, Japan) and the protocol from an earlier report [17]. In brief, 5,000 rabbit skin fibroblast cells were purchased from Jining Biotech Corp. Shanghai and cultured in high-glucose Dulbecco’s modified Eagle medium (DMEM) with 10 % fetal bovine serum (FBS). Then, they were were seeded in 96-well plates and treated with or without AAN-II (1 µM). After treatment, 10 µl of CCK-8 solution was added. After 1 h, the absorbance at 450 nm was measured using a microplate reader (Bio-Rad, USA).

### NGS-based transcriptome analysis

Total RNA from tissues with or without AAN-II treatment was extracted using Trizol Reagent (Invitrogen, USA) and cDNA was synthesized using First-strand cDNA synthesis kit according to the manufacturers’ protocols (Roche Applied Science, USA). The cDNA was sequenced at 1 × 100 bp/single read using an Illumina HiSeq 3000 instrument (Illumina, USA). Then, the obtained sequences were blasted with the set of chromosomes of the rabbit NCBI project 12,819, AAGW00000000 assembly, accessible at the public UCSC Genome Bioinformatics Site (http://genome.ucsc.edu/). The differentially expressed genes were verified with a threshold for absolute fold change > 1.5 and for false discovery rate (FDR) < 0.05. IPA analysis was used to define the enriched annotational functions. An absolute value > 2 is considered significant.

### CRISP-cas9 and cell transfection

To effectively downregulate TGF-β expression, two sgRNA target sequences were synthesized (5′-CGTACTTGTTTACACCCATG-3′ and 5′-ACAAGTTGACGGGACAGAAG-3′) for the TGFB1 gene. A non-silencing sequence (CGCTTCCGCGGC CCGTTCAA) was used as a negative control. Synthesized constructs were cloned into a lenti-CAS9-sgRNA-EGFP vector with an XbaI site and then transfected into 293 T cells using Lipofectamine 3000 according to the manufacturer’s instructions (Invitrogen, USA). The lentivirus particles with TGFB1-sgRNA were collected for further use. Subsequently, rabbit skin fibroblast cells were seeded into 6-well plates and transfected with the lentivirus and polybrene. Cell extracts were collected for molecular tests at the indicated times.

### Western blotting

Treated cells were lyzed using RIPA lysis buffer containing 1 mM phenylmethylsulfonyl fluoride (PMSF). Protein extracts were quantified using a Bio-Rad protein assay (Bio-Rad Laboratories, USA). Electrophoresis was performed on 20 µg protein samples on a 10 % SDS-polyacrylamide gel, followed by transfer to a 0.2 μm PVDF membrane using a Bio-Rad semi-dry instrument. After blocking with 5 % BSA in TBST buffer for 1 h at room temperature, the membranes were incubated overnight at 4 °C with various primary antibodies including TGF-β (1:1000), total or phosph-Smad3 (1:1000) and Smad7 (1:1000) from Santa Cruz Biotechnology Company. β-actin was the internal reference. After incubation with an anti-goat secondary antibody, the membranes were subjected to ECL western blot system (Pierce, USA) according to the manufacturer’s instructions. Quantification of bands was analyzed using QuantityOne software (Bio-Rad Laboratories, USA).

### ELISA assay

The filtered supernatant and appropriate ELISA kits were used to determine the levels of various cytokines according to the manufacturer’s instructions (R&D Systems, USA) and the protocol from an earlier report [18].

### Statistical analysis

Data were analyzed using statistical analysis software (SPSS 19.0, USA) and statistical mapping was performed using GraphPad Prism 11.0. Quantitative variables were determined using a one-way analysis of variance (ANOVA). Student’s t-test was used for two-group comparisons. *p* < 0.05 is considered statistically significant.

## Results

### AAN-II facilitates ulcer healing by suppressing the development of inflammation in this model

**Fig. 1:**
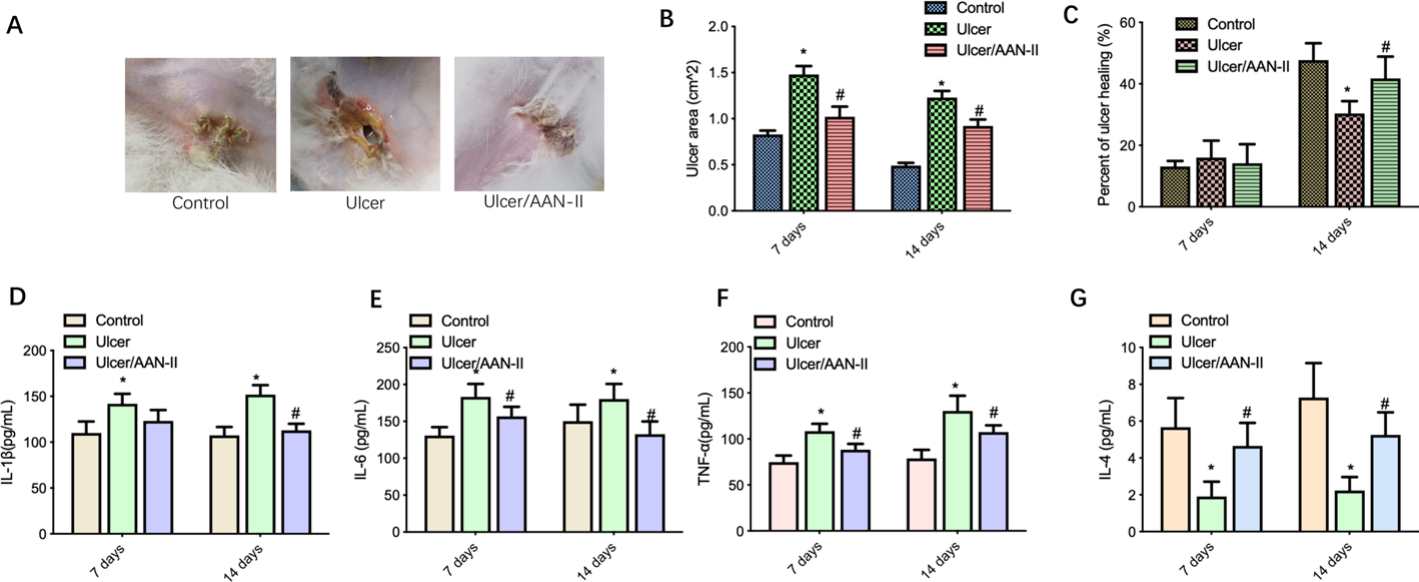
AAN-II facilitates ulcer healing by suppressing the development of inflammation in pressure-induced venous ulcers in a rabbit model. **A** Gross observation was performed to determine the pathological skin changes in the three groups. Representative images are shown (n = 10). **B** The ulcer area (cm^2^) was calculated on days 7 and 14 and the values are expressed as means ± SEM. *p < 0.05 compared with the control group. ^#^*p* < 0.05 compared with the ulcer group. **C** Percentage of ulcer healing was calculated on days 7 and 14 and the values are expressed as means ± SEM. *p < 0.05 compared with the control group. ^#^*p* < 0.05 compared with the ulcer group. **D** through **G **The levels of IL-1β (D), IL-6 (E), TNF-α (**F**) and IL-4 (**G**) were determined using ELISA kits. Values are expressed as means ± SEM. *p < 0.05 compared with the control group. ^#^*p* < 0.05 compared with the ulcer group

To determine whether AAN-II promotes ulcer healing in the pressure-induced venous ulcers in this rabbit model, we performed a histological analysis of ulcer wound morphology in the two ulcer groups. The results reveal that the re-epithelialization rate of the wounds was 38 % in the AAN-II treatment group, but only 16 % in the group without treatment (also as a positive control, similarly hereinafter). The healing of the wound-closure area was significantly faster in the AAN-II treatment group than in the group without treatment, in which the epithelium layer remained open and was covered by a large scab (Fig. 1A–C).

Cytokines are well-known regulators of the wound-healing process thanks to their promotion of angiogenesis and recruitment of inflammatory cells [19]. To investigate possible changes in chemokines during wound healing with AAN-II treatment, we analyzed the overall features of cytokines, including IL-1β, IL-6, TNF-α and IL-4, in all three groups on days 7 and 14 (Fig. 1D–G). The results show that compared to the control group, the ulcer group presented an increase in the levels of pro-inflammatory cytokines, including IL-1β, IL-6 and TNF-α, and a decrease in the level of anti-inflammatory cytokine IL-4 AAN-II treatment significantly induced a decrease in pro-inflammatory cytokine levels and an increase in the anti-inflammatory cytokine level.

### AAN-II promotes proliferation of fibroblasts and secretion of proangiogenic factors

**Fig. 2:**
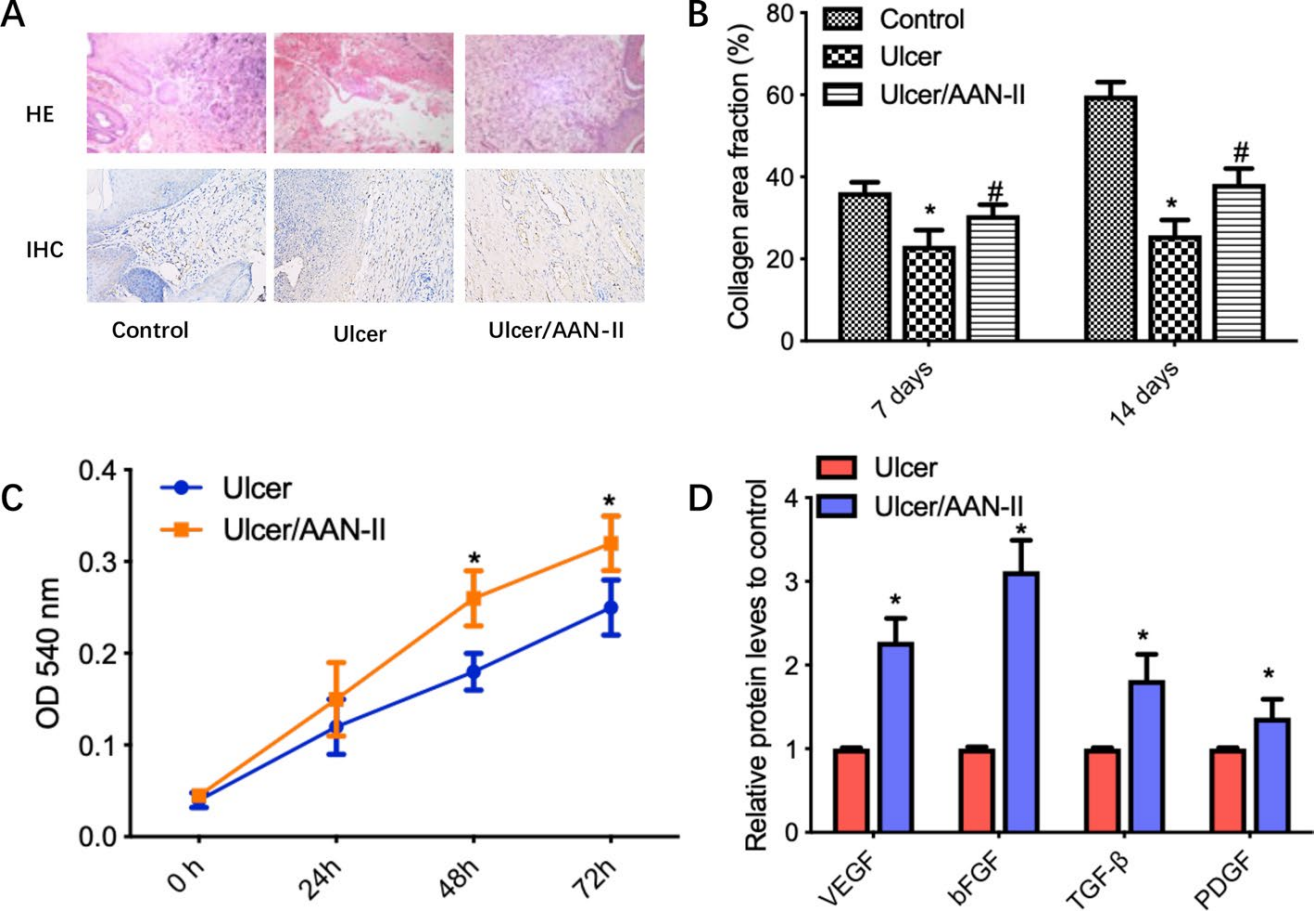
AAN-II promotes fibroblast proliferation and secretion of proangiogenic factors. **A** Hematoxylin and eosin (HE) staining was used to determine the skin pathological changes and immunohistochemical staining with anti-vimentin antibody was performed in the three groups. Representative fields are shown (×200). **B** The collagen area fraction (%) was calculated on days 7 and 14 and values are expressed as means ± SEM. *p < 0.05 compared with the control group. ^#^*p* < 0.05 compared with the ulcer group. **C** CCK-8 assay was used to determine the proliferation ability of rabbit skin fibroblast cells with or without AAN-II treatment. Values are expressed as means ± SEM. *p < 0.05 compared with the control group. **D** The relative protein levels of the proangiogenic factors VEGF, bFGF, TGF-β and PDGF were measured using ELISA assays. Values are expressed as means ± SEM. *p < 0.05 compared with the control group

A key step in healing is the transition from inflammation to cell proliferation [20]. Recent works have showed that fibroblasts play a role in the inflammation-to-proliferation transition and are critical in the deposition and remodeling of extracellular matrix components and wound contraction [21]. In the study, the ulcer groups with and without AAN-II treatment were compared. The results show that the number of fibroblasts significantly increased after AAN-II treatment (Fig. 2A), suggesting an obvious proliferation of fibroblasts during ulcer healing. The collagen fraction was significantly reduced on days 7 and 14 in the ulcer group compared to the control group, while the collagen fraction gradually improved in the group with AAN-II treatment compared with the group without treatment (Fig. 2B).

Subsequently, to demonstrate the effects of AAN-II on fibroblast proliferation, fibroblasts were cultured in vitro and treated with AAN-II. The results from the CCK-8 assay show that after AAN-II treatment, cell viability significantly increased compared to untreated cells (*p* < 0.05; Fig. 2C). Moreover, the levels of some cytokines that promote the proliferation of fibroblasts, including VEGF, bFGF, TGF-β and PDGF, significantly increased in the AAN-II treated cells compared with the untreated cells (*p* < 0.05; Fig. 2D). These results suggest that AAN-II could promote fibroblast proliferation during ulcer healing.

### AAN-II enhances the activation of the TGF-β1/SMADs signaling pathway

To explore the molecular mechanism of AAN-II promoting fibroblast proliferation, transcriptome sequencing technology was utilized to obtain the mRNA expression profiles with or without AAN-II treatment. Comparisons, bioinformatics analyses and validation experiments were then conducted.

**Fig. 3:**
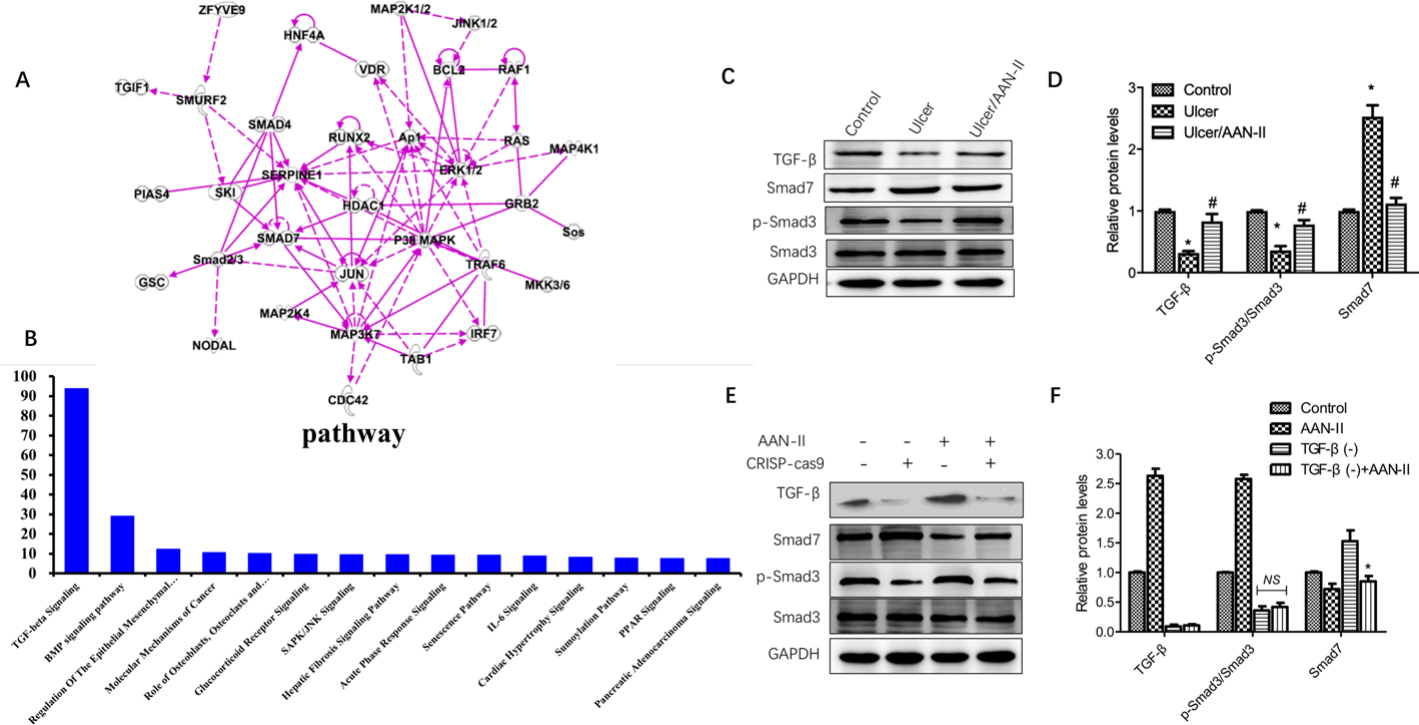
AAN-II enhances activation of the TGF-β1/SMADs signaling pathways during ulcer healing. **A** The IPA analysis of AAN-II treatment-related DEG interaction network. B – Gene ontology analysis was done using a bioinformatics-based approach. **C**, **D** TGF-β1 and its downstream proteins p-Smad3, Smad3 and Smad7 were measured via western blotting in different groups. Data are representative of 3 independent experiments, shown as ratios of these proteins to GAPDH and presented as means ± SEM. *p < 0.05 compared with the control group. ^#^*p* < 0.05 compared with the ulcer group. **E**, **F **TGF-β1 and its downstream proteins p-Smad3, Smad3 and Smad7 were measured via western blotting in different groups with or without TGF-β knockdown or AAN-II treatment. Data are representative of 3 independent experiments, shown as ratios of these proteins to GAPDH and presented as means ± SEM. *p < 0.05 compared with the TGF-β knockdown group

The results show that there 212 mRNA had different expressions between the treated and untreated groups. These dysregulated mRNAs mainly function in metabolic and repair processes and participate in 32 biological pathways, with the TGF-β1/SMADs signaling pathway at #1 (Fig. 3A, B).

Therefore, the protein levels of some TGF-β1/SMADs-related molecules, including TGF-β, Smad7, phosphor-Smad3 and total Smad3, were determined in the purified primary fibroblasts from the control, ulcer and ulcer with AAN-II treatment groups. The levels of TGF-β and phosphor-Smad3/Smad3 were significantly lower in the ulcer group than in the control group, but slightly higher in the AAN-II treatment group. The levels of Smad7 in the three groups showed the opposite change (Fig. 3C, D).

To further investigate whether the role of AAN-II is dependent on the presence of TGF-β, we knocked out TGF-β in fibroblasts with a CRISPR-Cas 9 system and determined the protein levels of TGF-β, Smad7, phosphor-Smad3, and total Smad3. When TGF-β was knocked out, AAN-II treatment could not significantly upregulate phosphor-Smad3 or total Smad3 and the level of smad7 decreased (Fig. 3E, F), suggesting a dependence of AAN-II on the activation of TGF-β/smad3.

### AAN-II and TGF-β present synergistic effects on ulcer healing in this rabbit model

**Fig. 4:**
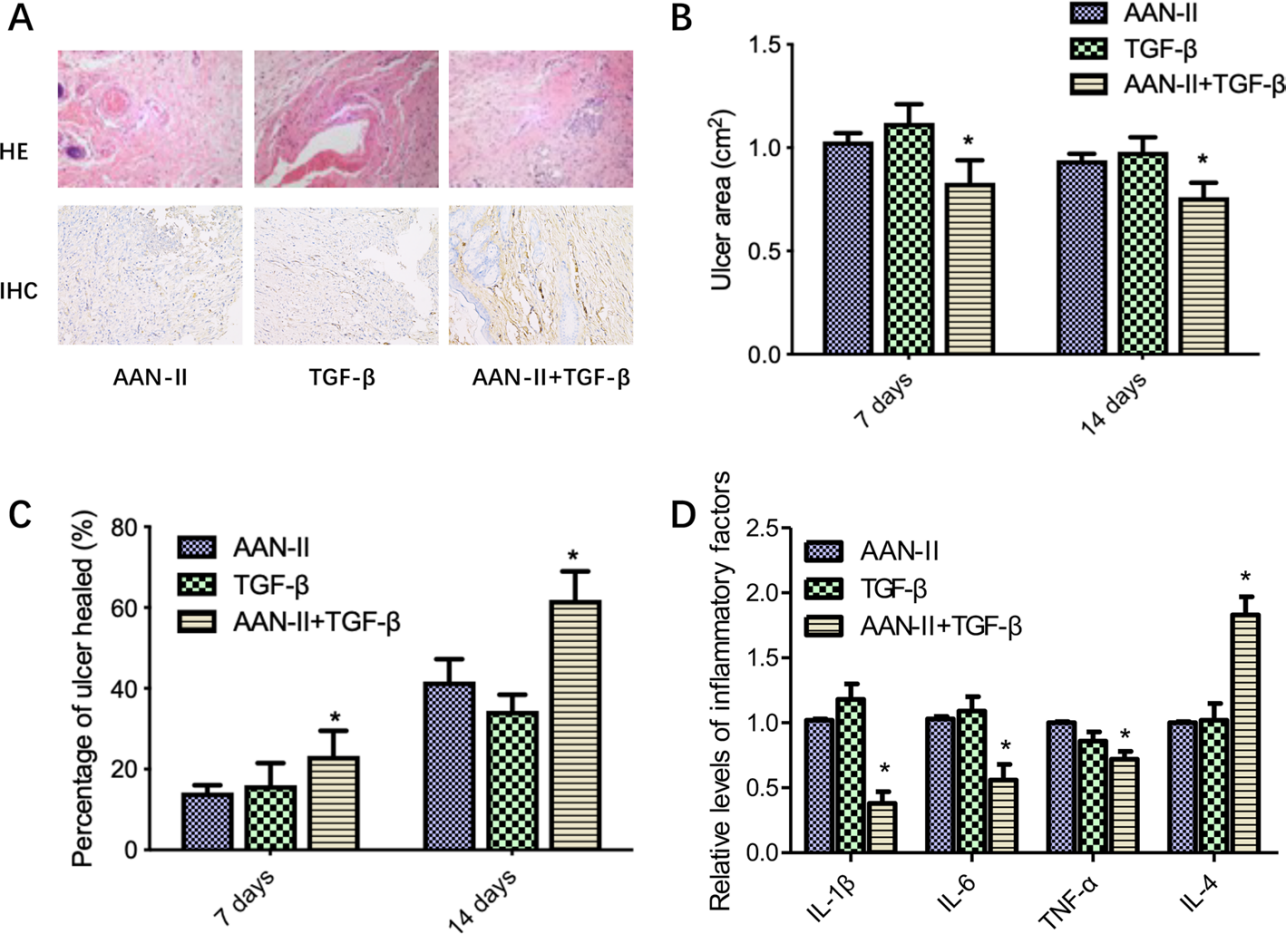
AAN-II and TGF-β present synergistic effects on ulcer healing in model animals. **A** Hematoxylin and eosin (HE) staining was used to determine the skin pathological changes and immunohistochemical staining with anti-vimentin antibody was performed in the three ulcer groups respectively treated with AAN-II, TGF-β and a combination of AAN-II and TGF-β. Representative fields are shown. **B** Ulcer area (cm^2^) was calculated on days 7 and 14 and values are expressed as means ± SEM. *p < 0.05 compared with the AAN-II group. **C** Percentage of ulcer healed was added up on days 7 and 14 and values are expressed as means ± SEM. *p < 0.05 compared with the AAN-II group. **D** The levels of IL-1β, IL-6, TNF-α and IL-4 were determined using ELISA kits. Values are expressed as means ± SEM. *p < 0.05

To investigate whether AAN-II and TGF-β can synergistically improve ulcer healing, the ulcers in the model animals were independently treated with AAN-II or TGF-β or a combination of AAN-II and TGF-β. Pathohistochemical analysis showed that the combination of AAN-II and TGF-β significantly promoted significantly the proliferation of fibroblasts (Fig. 4A), reduced the ulcer area (Fig. 4B) and increased the percentage of ulcer healing (Fig. 4C) compared with the effects of AAN-II or TFG-β alone. Furthermore, the relative levels of inflammatory factors including IL-1β, IL-6 and TNF-α were significantly lower and the IL-4 level was higher (Fig. 4D). These results indicate that AAN-II and TGF-β present synergistic effects on ulcer healing in this rabbit model.

## Discussion

Histopathological analysis confirmed the successful establishment of our rabbit model of venous leg ulcers (VLUs). When compared, the ulcer group without treatment had higher proliferation rates for inflammatory cells and fibrous connective tissue and the control group showed low levels of inflammatory cell infiltration and necrotic neutrophils. Our model provided a solid experimental platform for our subsequent experiments.

The current important issues in the management of VLUs in the lower limbs are professional uncertainty and clinical variability [22]. The therapeutic pharmacology for VLUs mainly involves two medications: pentoxifylline and phlebotropic agents [23]. Traditional Chinese medicine is becoming more widely accepted in the treatment of chronic ulcers. For example, a meta-analysis showed, compared with other treatments, effects of Chinese herbal medicine ointment for pressure ulcer on the total effective rate are beneficial [8]. Traditional Chinese medicine assumes that chronic skin ulcers always have “virtual” and “stasis” states because of the incessant healing. In previous studies, we observed wound healing after external use of *Zizhu* herbal ointment (a traditional Chinese herbal formulation from Shuguang Hospital Affiliated to Shanghai University of Traditional Chinese Medicine), in patients with diabetic foot ulcers or VLUs in clinical practice [24–26]. *Alkanna tinctoria* is the main component of this formulation, and it has been identified as effective in the treatment of dermatitis, including psoriasis [27].

Alkannin is an important bioactive component of *A. tinctoria* with four bioactive components: AAN-I, AAN-II, AN-III and AN-IV. We focused on AAN-II, finding that it facilitates ulcer healing by suppressing the development of inflammation. We demonstrated an AAN-II treatment-induced decrease in pro-inflammatory cytokine levels and an increase in anti-inflammatory cytokine levels. These phenomena could be related to inhibition of AAN-II in elevated venous pressure-induced inflammatory cascades.

Healing is a multistep process, involving hemostasis, proliferation, inflammation, immunological response and remodeling [19, 28]. It is well recognized that fibroblasts are critical in supporting wound healing [1]. Therefore, we evaluated the proliferation of fibroblasts after AAN-II treatment and verified that it could promote this process during the ulcer healing. We also identified the TGF-β/SMADs signaling pathway as the crucial node among the AAN-II-related networks using transcriptome sequencing technology combined with bioinformatics analyses. The effects of AAN-II were shown to be dependent on the activation of TGF-β/smad3 signaling.

This is the first demonstration of the association between AAN-II and TGF-β/smad3 signaling. Developing novel therapeutic approaches requires a better understanding of the mechanisms that underly wound healing [29]. Therefore, we further explored the therapeutic potential of targeting the chemokine TGF-β and using AAN-II together as a novel approach for VLU treatment. Intriguingly, we found that they presented synergistic effects on ulcer healing.

## Conclusions

These results demonstrate the effects of AAN-II on inhibiting inflammation and promoting fibroblast proliferation in this model. Additionally, our in vivo results demonstrate that AAN-II and TGF-β had synergistic effects on ulcer healing. These findings might provide research evidence for a novel therapeutic approach to venous leg ulcers or venous ulcers in general. Clinical research is necessary to determine the feasibility and therapeutic benefit of AAN-II for ulcer healing.

## Data Availability

The datasets used and/or analyzed during the current study are available from the corresponding author on reasonable request.

## Abbreviations

ANN: Alkannin
bFGF: Basic fibroblast growth factor
CCK-8: Cell Counting Kit-8
DMEM: Dulbecco’s modified Eagle medium
FBS: Fetal bovine serum
FFPE: Formalin-fixed paraffin-embedded
H_2_O_2_: Hydrogen peroxide
HE: Hematoxylin and eosin
HSP70: Heat shock protein 70
IHC: Immunohistochemistry
IL-1β: Interleukin-1β
IL-4: Interleukin-4
IL-6: Interleukin-6
PMSF: Phenylmethylsulfonyl fluoride
PVDF: Polyvinylidene fluoride
PBS: Phosphate-buffered saline
PDGF: Platelet-derived growth factor
RIPA: Radio Immunoprecipitation assay
TBST: Tris-buffered saline Tween
TGF-β: Transforming growth factor-β
TNF-α: Tumor necrosis factor-α
UVB: Ultraviolet radiation
VEGF: Vascular endothelial growth factor
VLU: Venous leg ulcer
